# Modeling pegcetacoplan treatment effect for atrophic age-related macular degeneration with AI-based progression prediction

**DOI:** 10.1186/s40942-025-00634-z

**Published:** 2025-02-07

**Authors:** Irmela Mantel, Romina M. Lasagni Vitar, Sandro De Zanet

**Affiliations:** 1 Department of Ophthalmology, University of Lausanne, Jules-Gonin Eye Hospital, Fondation Asile des Aveugles, Lausanne, Switzerland; 2 Medical Faculty, University of Lausanne, Lausanne, Switzerland; 3 Ikerian AG, Freiburgstrasse 3, Bern, 3010 Switzerland

**Keywords:** Age-related macular degeneration, Atrophy, Complement inhibition, Pegcetacoplan, Deep learning-based RORA progression prediction

## Abstract

**Background:**

To illustrate the treatment effect of Pegcetacoplan for atrophy secondary to age-related macular degeneration (AMD), on an individualized topographic progression prediction basis, using a deep learning model.

**Methods:**

Patients (*N* = 99) with atrophy secondary to AMD with longitudinal optical coherence tomography (OCT) data were retrospectively analyzed. We used a previously published deep-learning-based atrophy progression prediction algorithm to predict the 2-year atrophy progression, including the topographic likelihood of future retinal pigment epithelial and outer retinal atrophy (RORA), according to the baseline OCT input. The algorithm output was a step-less individualized topographic modeling of the RORA growth, allowing for illustrating the progression line corresponding to an 80% growth compared to the natural course of 100% growth.

**Results:**

The treatment effect of Pegcetacoplan was illustrated as the line when 80% of the growth is reached in this continuous model. Besides the well-known variability of atrophy growth rate, our results showed unequal growth according to the fundus location. It became evident that this difference is of potential functional interest for patient outcomes.

**Conclusions:**

This model based on an 80% growth of RORA after two years illustrates the variable effect of treatment with Pegcetacoplan according to the individual situation, supporting personalized medical care.

## Background

Age-related macular degeneration (AMD) in its advanced form is the leading cause of irreversible visual impairment and legal blindness worldwide [1]. Advanced stages include neovascular AMD (nAMD) and geographic atrophy (GA, also called non-neovascular advanced AMD) [2]. While the neovascular form of late AMD has been treatable for nearly 2 decades with intravitreal anti-VEGF injections [3–6], the first treatment options for the atrophic form of late AMD have only recently been approved by the Food and Drug Administration (FDA).

Pegcetacoplan was the first drug to be approved. It is a C3 Complement inhibitor that has reached the primary endpoint in phase 3 pivotal trials OAKS and DERBY, for both, fixed monthly or every other month intravitreal injections [7]. Atrophic lesion growth significantly decreased by 20% on average after two years of monthly treatment compared with sham injections. Interestingly, a subgroup analysis of the change in lesion size by lesion location revealed that the treatment effect was significantly higher after 12 and 24 months in patients presenting extrafoveal lesions at baseline when compared with sham injections (12 months: extrafoveal, 26%; subfoveal, 11%; 24 months: extrafoveal, 26%; subfoveal, 19%). Other subgroup analyses showed no significant differences.

Even though these findings are very encouraging, it remains difficult to understand what a 20% growth reduction means for a given patient, since progression rates can significantly vary among patients (slow vs. rapid progression), ranging from 0.53 to 2.6 mm^2^/year (median 1.78 mm^2^/year) [8]. In this scenario, a 20% reduction of the lesion growth may have a low impact on slow progressor patients, whilst on rapid progressors this reduction may be more meaningful in terms of structural and functional outcomes. Moreover, the progression rate may vary across the fundus when lesions are topographically unequally distributed (proximity to the fovea) and different severity stages and extensions of the preceding outer retinal disruption are observed, as previously shown by others [9].

We recently developed a deep learning-based algorithm for the progression prediction of retinal pigment epithelial and outer retinal atrophy (RORA) over 5 years, applicable on a single OCT volume (RetinAI GA Progression Prediction, Ikerian AG, Bern, Switzerland). The algorithm was trained and validated with longitudinal OCT volume data from atrophic AMD patients [10]. The algorithm achieved good predictive performance for atrophy growth over 5 years. Its particular advantage includes a continuous progression scale, allowing for an individualized en-face atrophy risk map, established based on a single OCT scan volume at baseline.

As our progression prediction algorithm was able to predict atrophy growth at any time point (continuous), it was the ideal basis for evaluating the line at which the 80% of natural growth of 2 years was reached. With this investigation, we aimed to illustrate what the 20% growth reduction under treatment with Pegcetacoplan would mean in terms of spared atrophy growth in individual patients.

## Methods

### Study design and data collection

This is a retrospective image analysis study, using data from the training set of a previously published deep-learning-based algorithm development. The training data was described in detail in the previous publication [10]. Briefly, it included longitudinal data from retinal OCT images retrospectively collected from patients with atrophy secondary to AMD (GA) at the Hospital Ophthalmic Jules-Gonin (Lausanne, Switzerland). In total, we included longitudinal OCT data from 99 patients (109 eyes) with at least one follow-up visit (1–7 years follow-up), and at least two OCT examinations, acquired with a Heidelberg Spectralis OCT device (Heidelberg Engineering, Heidelberg, Germany). The OCT scans were routinely acquired with a macular cube of 6 × 6 mm, 49 B-scan lines or more, and using the inbuilt follow-up mode for subsequent visits. We excluded patients showing neovascular complications or with a history of anti-VEGF treatment, confounding retinopathy, as well as poor-quality images. Before analysis, all data were fully coded, eliminating all personal patient information.

For the present publication, the resulting algorithm was applied to the baseline OCT images from these training data. The method of determining the growth areas is described below.

The study was approved by the local ethics committee (CER-VD 2017 − 00493) and was performed according to the ethical standards set by the Declaration of Helsinki. No informed consent was required. Clinical trial number: not applicable.

### Artificial intelligence-based OCT analysis

To analyze the presence of RORA and to predict its progression over time, we used a recently developed deep learning-based algorithm (RetinAI Medical AG, Bern, Switzerland), trained and validated with longitudinal OCT volume data from atrophic AMD patients [10].

Briefly, OCT volumes were uploaded through the RetinAI Discovery platform (Ikerian AG, Bern, Switzerland) and pre-processed to obtain automated segmentations of retinal layers and Drusen [11]. Subsequently, enface thickness and mean reflectivity maps were obtained and used as the input to the Convolutional Neural Network of RORA progression prediction algorithm. Atrophy prediction over time was visualized in en-face projections.

As we aimed to illustrate the impact of the overall 20% lesion growth reduction resulting from Pegcetacoplan treatment compared with the natural growth over two years, we evaluated a series of patients from the training data used to develop the algorithm. A range of different clinical situations was selected, all according to the inclusion criteria of the pivotal trials showing the efficacy of Pegcetacoplan. These inclusion criteria were: ≥2.5 and ≤ 17.5 mm^2^ total atrophy size; If multifocal, at least on atrophy patch ≥ 1.25 mm^2^; perilesional hyper-autofluorescence; both subfoveal and non-subfoveal location were allowed.

The algorithm provided step-less topographic modeling of the RORA growth, thus allowing for a calculation of the progression line corresponding to an 80% growth compared to the natural course of 100% growth. Results were shown as three-line-illustration in *en face* maps: baseline RORA extension, 80% growth at 2 years, and 100% growth (natural course) at 2 years.

## Results

To illustrate the impact of Pegcetacoplan’s treatment effect on different clinical situations, we performed a retrospective analysis of longitudinal OCT data of patients presenting atrophy secondary to AMD. The demographics of the study population are presented in Table 1.

**Table 1:** Patients’ demographics

Characteristic	Dataset
Number of patients	99
Number of eyes	109
Sex (%, male/female)	31.3/ 68.7
RORA area at BL (mm^2^)^*^	4.2 ± 4.5
Follow-up time (months)^*^	35 ± 26
Growth rate (mm/year)^*^	0.34 ± 0.23

^*^Results are expressed as mean ± standard deviation. RORA: retinal pigment epithelial and outer retinal atrophy; BL: baseline

**Fig. 1:**
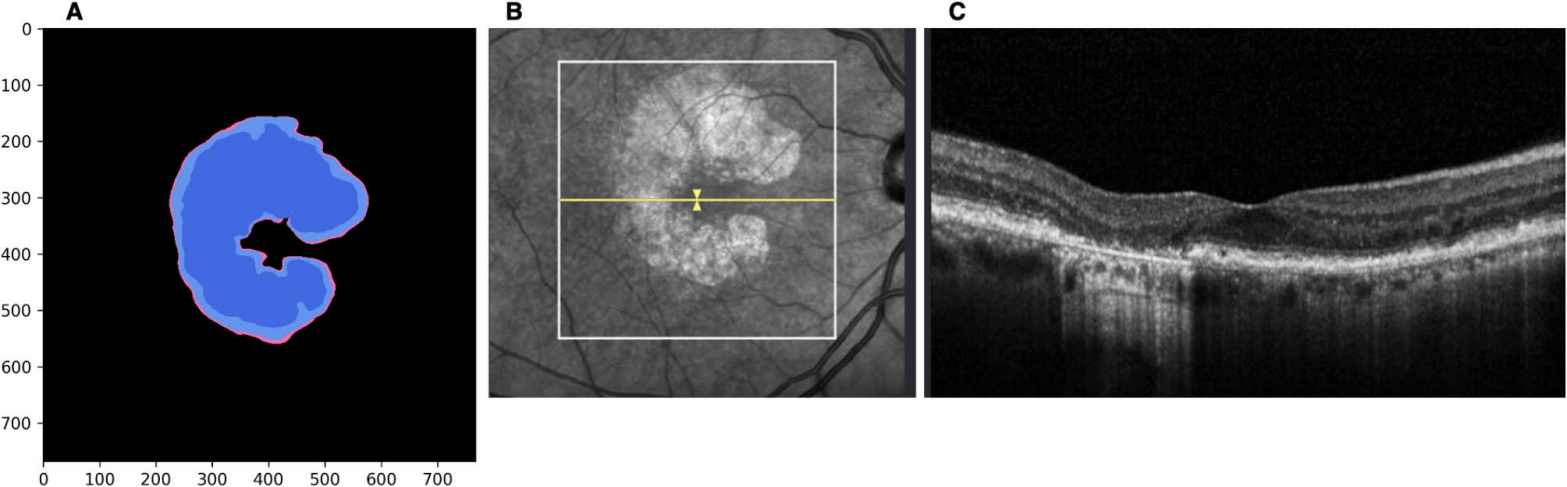
Pegcetacoplan treatment effect model. (**A**) The progression prediction algorithm provides a three-line illustration in en-face maps: retinal pigment epithelial and outer retinal atrophy (RORA) area at baseline (dark blue), 80% lesion growth at 2 years (light blue), and 100% growth (natural course) at 2 years (pink). The pink area corresponds to the calculated treatment effect (20% growth reduction). (**B**) En-face projection of segmented RORA area shown with the localizer (in yellow). The foveal location is indicated by the position of the localizer. (**C**) Corresponding B-scan

An example of modeling the treatment effect is shown in Fig. 1. The AI-based progression prediction provided a three-line illustration in en-face maps: RORA area at baseline (dark blue), 80% growth at 2 years (light blue), and 100% growth (natural course) at 2 years (pink). The pink area represents the predicted 20% growth reduction resulting from the Pagcetacoplan treatment over 2 years.

**Fig. 2:**
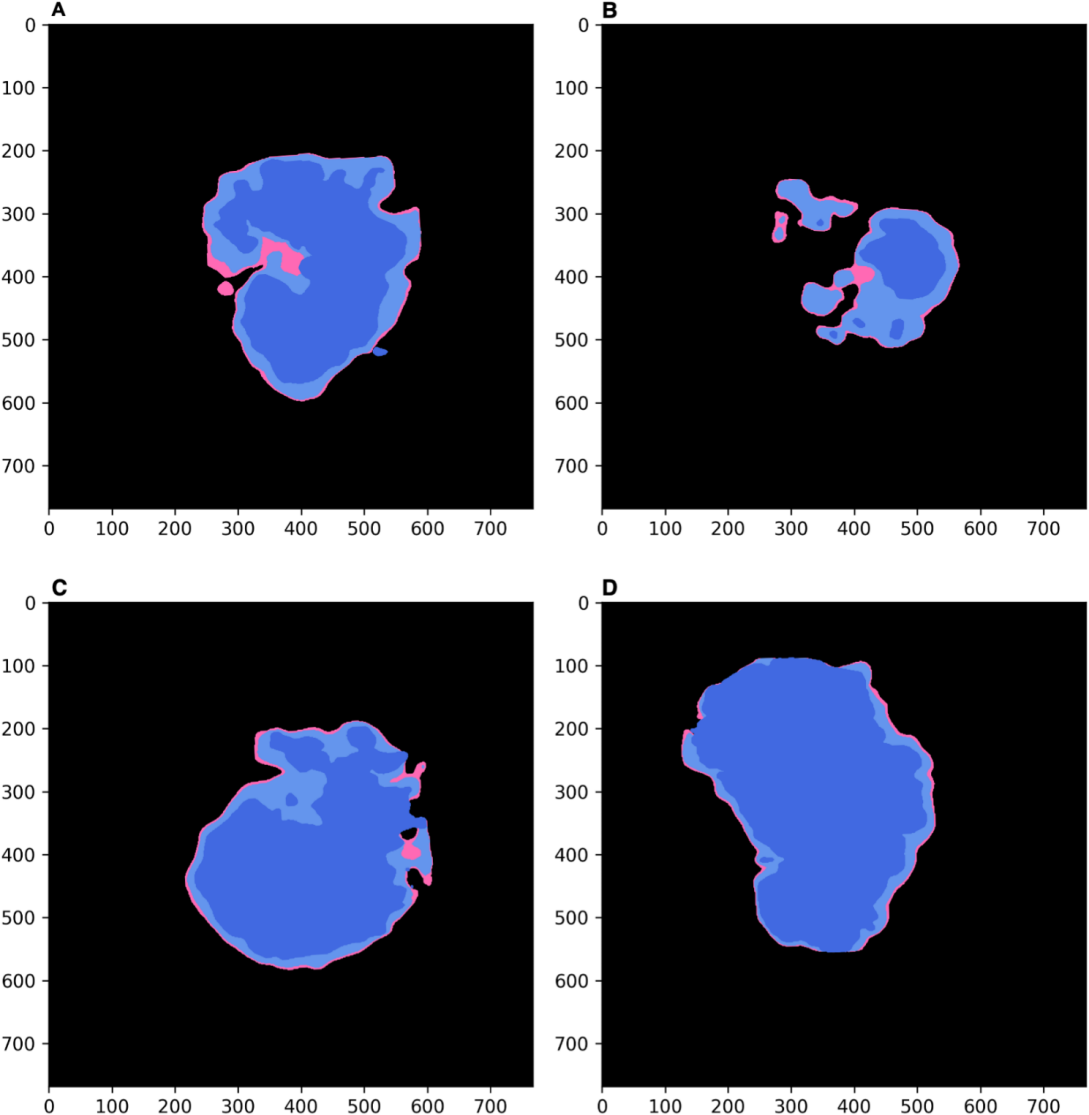
Prediction of treatment benefits on an individualized basis. The interest in treatment becomes more evident when the lesion is predicted to evolve towards the fovea (**A** and **B**), whilst it can be limited when the lesion is already central and the fovea is compromised from baseline (**C** and **D**)

In line with the well-known variability of atrophy growth rate, our results showed unequal growth according to the lesion size at baseline and fundus location (Fig. 2). When modeling the treatment effect on an individual basis, it became evident that the variability accompanying atrophy lesion growth impacts the treatment benefit, especially when the lesion is predicted to evolve towards the fovea (Fig. 2, A and B). However, the effect seemed to be limited when the lesion was already central and the fovea was compromised from baseline (Fig. 2, C and D).

## Discussion

In this study, we aimed to illustrate the treatment effect of Pegcetacoplan for atrophy secondary to AMD, on an individualized basis, using AI-based topographic progression prediction of the atrophic lesion. Recently, clinical trials have demonstrated that the statistical mean effect of monthly injections of Pegcetacoplan was a 20% lesion growth reduction in the entire cohort. Based on the subgroup analyses conducted, the relative efficacy appears consistent across patients, except for variations associated with lesion location (foveal versus extrafoveal). Nevertheless, clinical observation readily reveals that the significance of this treatment effect varies among individual patients. In this regard, it is well-known that the atrophy progression rate is significantly dependent on the location of the lesion with respect to the fovea and the topography of the growth [12]. In line, our results showed unequal growth according to the fundus location of the lesion and, thus, it becomes evident that this difference is of variable interest to the treatment outcome.

In addition, the lesion growth reduction only indirectly translates into visual function status, as not only the RORA area is non-functional, but also the surrounding area shows abnormal function [13]. Thus, it is challenging to translate the atrophy en-face maps into an idea of visual function, the ultimate interest of the patient. However, the representation of atrophy progression allows for an accurate illustration of the relative effect.

Interestingly, the pivotal trials OAKS and DERBY did not show a visual benefit in terms of central visual acuity [7]. This is not surprising, as this is mostly dependent on the foveal state, for which atrophy is only one of the relevant features to consider in the foveal area. In addition, extrafoveal atrophy location does not impact the central visual acuity. In this vein, it is generally admitted that measuring central visual acuity is an inappropriate outcome measure for demonstrating functional limitations due to atrophy and its growth [14, 15]. In this scenario, microperimetry was suggested to overcome some of the limitations linked to central visual acuity tests [16]. The post hoc analysis of data from the OAKS trial revealed that Pegcetacoplan showed a reduced number of scotomatous points within the junctional zone of the atrophic lesion, but only after a 2-year treatment [7]. We speculate that this might occur because microperimetry is not densely sampled and the samples themselves are noisy, thus missing minor surface changes in atrophy. A longer-term evaluation over 2 years would probably better reflect the observed changes.

Recently, it has been shown that the loss of photoreceptor layer integrity around the GA lesion is a relevant parameter for atrophy growth and also for treatment effect with Pegcetacoplan [17]. Therefore, the complete atrophy criterion may not be the most appropriate measure of the functionally relevant treatment effect, as prior photoreceptor damage is likely to be associated with preceding functional disturbances. However, complete atrophy corresponds to the clinically best visible lesion and clinicians usually focus on complete atrophy as the relevant lesion on the fundus. Consequently, while targeting earlier stages rather than complete atrophy may be functionally more relevant, our model could assist clinicians in understanding the treatment effects at this advanced stage.

The complete atrophy lesion growth and the loss of photoreceptor integrity are now considered outcome measures that are relevant for the patient and accepted by the FDA for clinical trials. Indeed, both types of lesion size and location ultimately affect the remaining visual function of the eye.

An interesting observation in our study was that extrafoveal lesions tended to show more treatment effect in the foveal area than outside the fovea. This corresponded well with a post hoc analysis of the OAKS and DERBY studies showing that growth towards the fovea was better protected in the treatment arms than other growth [18]. The protective effect on the foveal zone would have been even more pronounced if the relatively greater treatment effect observed in the extrafoveal lesion subgroup (26% at 2 years) had been applied for modeling [7].

Our analysis comes with some limitations. First, the model relies on 2-year data from the pivotal trial as a reference point. It is evident that the relative significance of the treatment effect could vary if a different endpoint were used. Unfortunately, long-term data are yet not available.

Estimating the treatment effect over an extended period is further complicated by the fact that participants in the sham arm eventually received treatment if they continued in the trial. An interesting alternative would involve training a similar algorithm using data from the treatment arm and comparing AI predictions with and without treatment.

Second, the AI-based models inherently include a margin of error. However, in this study this error was minimized by employing the training data itself, thereby ensuring the highest possible precision in prediction outcomes between input time points. The manually annotated growth pattern was regularized by the machine learning model to produce a smooth, time-dependent growth pattern.

Finally, the number of cases included may not cover the full spectrum of atrophic AMD. Nevertheless, the selection process was designed to include as diverse a range of cases as possible within the constraints of the available data.

## Conclusion

In summary, we presented an automated way of modeling the treatment effect of a drug for atrophic AMD, based on an individualized topographic progression prediction basis, using a deep learning model. Although we do not have a progression model based on a treated cohort, this model accurately illustrates the variable geographic effect of treatment according to the individual situation, based on an 80% growth of RORA after 2 years. Our model may help clinicians easily understand and visualize treatment benefits for a given patient, allowing for personalized medical care.

## Data Availability

The datasets generated and/or analysed during the current study are not publicly available due to privacy or ethical restrictions.

## Abbreviations

AI: Artificial intelligence
AMD: Age-related macular degeneration
FDA: Food and drug administration
GA: Geographic atrophy
nAMD: Neovascular AMD
OCT: Optical coherence tomography
RORA: Retinal pigment epithelial and outer retinal atrophy
